# Influence of Estradiol Levels and Menstrual Cycle Phase on Basal and Exercise-Induced ROS and IL-6 Responses in Eumenorrheic Women

**DOI:** 10.3390/sports14050168

**Published:** 2026-04-22

**Authors:** Markus Gassner, Johanna Diewald, Linda Leichtfried, Lucie Zeller, Serena Ryan, Karl-Heinz Wagner, Daniel König

**Affiliations:** 1 Faculty of Life Sciences, Department for Nutrition, Section for Nutrition, Exercise and Health, University of Vienna, Josef Holaubek Platz, 1090 Vienna, Austria; 2 Vienna Doctoral School of Pharmaceutical, Nutritional and Sport Sciences, University of Vienna, Josef Holaubek Platz, 1090 Vienna, Austria; 3 Centre for Sports Science and University Sports, University of Vienna, Josef Holaubek Platz, 1090 Vienna, Austria; 4 FH Campus Wien, University of Applied Sciences, 1100 Vienna, Austria; 5 Department of Nutritional Sciences, Research Platform Active Ageing, Josef Holaubek Platz, University of Vienna, 1090 Vienna, Austriakarl-heinz.wagner@univie.ac.at (K.-H.W.); 6 Centre for Sports Science and University Sports, Department of Sports Science, Section for Nutrition, Exercise and Health, University of Vienna, Auf der Schmelz, 1150 Vienna, Austria

**Keywords:** 17-beta-Estradiol, female, HIIT-induced oxidative stress, Interleukin-6, inflammation, exercise-induced oxidative stress, female adults, menstrual cycle

## Abstract

Biological differences between sexes—particularly due to fluctuating levels of 17β-estradiol and menstrual cycle dynamics—may influence exercise-induced reactive oxygen species (ROS) formation, inflammation and exercise performance. Despite these considerations, there is a lack of research exploring how estradiol and menstrual cycle phases may impact exercise performance, exercise-induced ROS formation and inflammation. This study aimed to examine whether estradiol concentration or menstrual cycle phase may be significantly associated with resistance circuit high-intensity interval training (HIIT) performance, as well as exercise-induced formation of ROS and Interleukin-6 (IL-6). A total of 30 young healthy female participants completed a single bout of resistance-based HIIT in a fasted state. Blood samples were collected at four time points: at baseline after overnight fasting, two hours after consumption of 0.5 L of water (pre-HIIT), immediately post exercise (post-HIIT) and after 15 min of recovery (15-post-HIIT). Additionally, participants attended six fasting baseline assessments scheduled across various menstrual cycle days. These sessions enabled the assessment of estradiol, ROS and IL-6 concentrations throughout the menstrual cycle without being confounded by nutritional factors. Neither baseline levels of ROS nor IL-6 differed significantly between menstrual cycle phases (luteal vs. follicular ROS: 0.013 µmol/min, *p* = 0.716; IL-6: 0.052, *p* = 0.679) menstruation status (yes vs. no ROS: −0.056 µmol/min, *p* = 0.259; IL-6: −0.302 pg/mL, *p* = 0.088) or 17β-estradiol concentrations (low (11–≤72.5 pg/mL) vs. high (>72.5–394 pg/mL) ROS: −0.038 µmol/min, *p* = 0.266; IL-6: +0.015 pg/mL, *p* = 0.906). On the resistance-circuit-HIIT intervention day, no significant differences in ROS or IL-6 were observed between estradiol concentrations (ROS: *p* = 0.477; IL-6: *p* = 0.249), menstrual cycle phase (ROS; *p* = 0.752; IL-6: *p* = 0.557) or menstruation status (ROS: *p* = 0.383; IL-6: *p* = 0.808) from baseline to pre-HIIT, post-HIIT or 15-post-HIIT. These findings should be interpreted with caution, as the menstrual cycle phases were assigned using a calendar-based approach without biochemical ovulation confirmation and the subgroup sizes were relatively small. These findings suggest that natural 17-beta-Estradiol fluctuations within the menstrual cycle, as well as differences in the menstrual cycle itself, may not substantially modulate ROS or IL-6 responses to acute resistance-based HIIT in young healthy female adults.

## 1. Introduction

Over recent decades, the majority of research has predominantly involved adult male participants [1], resulting in a persistent underrepresentation of women in exercise science. Female participants have often been considered more challenging to study, largely due to hormonal fluctuations across the menstrual cycle. However, these fluctuations may provide important physiological insights rather than limitations. In particular, the predominant female sex hormone, 17-beta-Estradiol, has been suggested to exert antioxidative properties [2,3] and to modulate inflammatory responses as well as tissue repair following physical activity [4]. Its antioxidative capacity is attributed to its phenolic hydroxyl group, which enables the scavenging of reactive oxygen species (ROS) [5]. During the reproductive years, estradiol (E2) is the most biologically active form of estrogen and can be reliably quantified in serum [2].

Exercise is known to induce oxidative stress through increased production of ROS, which can influence both cellular function and recovery processes. Notably, sex differences in oxidative stress responses have been reported. For instance, lipid peroxidation levels are significantly higher in post-pubertal boys compared to post-pubertal girls [6]. Similarly, young adult males exhibit higher biomarkers of oxidative stress than their female counterparts [7] and female vascular cells produce lower levels of ROS than male cells [8]. These observations suggest a potential protective role of female sex hormones, particularly estradiol, in modulating oxidative stress.

The menstrual cycle represents a major biological rhythm in females, second only to the circadian rhythm [9], and is characterized by substantial fluctuations in hormone levels, particularly estradiol. In adolescents, cycle length typically ranges from 21 to 45 days [10], and although a 28-day cycle is often used as a standard model, considerable interindividual variability exists [11]. The cycle is commonly divided into the pre-ovulatory (follicular) phase and the post-ovulatory (luteal) phase [12]. Estradiol concentrations are lowest during the early follicular phase, gradually increase, and peak approximately two to three days prior to ovulation [2]. These hormonal fluctuations may influence physiological responses to exercise, including oxidative stress and inflammation.

In recent years, increasing attention has been given to the potential impact of menstrual cycle phases on exercise performance and inflammatory responses. Some evidence suggests that high-intensity intermittent exercise during the luteal phase may modulate cytokine responses and enhance recovery, representing a potential non-pharmacological strategy to regulate inflammation [13]. A recent meta-analysis reported a tendency toward a higher pro-inflammatory response during the luteal phase [14]. Furthermore, reviews by Romero-Parra and Thompson [15,16] indicated reduced strength and increased delayed onset muscle soreness (DOMS) during the early follicular phase. However, other systematic reviews have found no consistent effect of menstrual cycle phase on strength performance [11,12,17,18]. These inconsistencies highlight the need for further investigation.

Despite growing interest in this field, the specific role of 17-beta-Estradiol, menstruation status and menstrual cycle phases in modulating exercise-induced oxidative stress, particularly in response to resistance-based high-intensity interval training (HIIT), remains insufficiently understood. While HIIT is an effective and widely used model for inducing physiological stress in both females and males [19,20], existing studies in young women have not adequately accounted for estradiol as a potential confounding factor. For example, a pilot study demonstrated increased ROS following HIIT in young female adults, but did not assess 17-beta-Estradiol levels [20].

Therefore, the present study aims to investigate the influence of key endogenous variables, namely 17-beta-Estradiol levels, menstruation status and menstrual cycle phase, on ROS production, IL-6 response and exercise performance following a resistance-based HIIT protocol in sedentary young women. By specifically focusing on female participants, this study seeks to address the longstanding underrepresentation of women in sports science research and to contribute to a more comprehensive understanding of sex-specific physiological responses to exercise [1,21,22].

## 2. Methods

### 2.1. Participants

A total of 42 healthy young female adults participated in this study. Participants were recruited through social media platforms. To be eligible for inclusion, individuals had to meet the following criteria: female sex, age between 18 and 40 years, absence of any acute or chronic illness and a body mass index (BMI) between 18.5 and 25 kg/m^2^. Exclusion criteria included smoking, engaging in more than two hours of regular physical activity per week, regular resistance training, any acute or chronic disease, use of medications, food allergies or intolerances, anemia, pregnancy or lactation.

Twelve participants who were using hormonal contraception were excluded from the final analysis due to its potential to be a significant confounding factor. Therefore, 30 participants were included in the final statistical analysis.

Each participant was required to visit the study center on seven occasions. For the cross-sectional part of this research project, during these visits, fasting morning baseline levels of 17β-estradiol, IL-6 and ROS were measured. The decision to perform all baseline measurements at fasted state was a deliberate methodological choice to minimize variability in metabolic and inflammatory markers that may be influenced by recent food intake [23]. This approach helps to ensure more standardized and comparable baseline measurements across participants. Additionally, data regarding each participant’s menstrual cycle was collected. On one of these visits, participants performed a single bout of resistance circuit-based HIIT in a fasted state, two hours after baseline assessments.

While designing this study, we deliberately chose an intensive baseline assessment phase with seven separate visits for fasting measurements paired with a single intervention day. The rationale for combining a cross-sectional analysis with an intervention day is rooted in the biological variability of our target population and the complexity of the biomarkers investigated. Female physiology is subject to dynamic fluctuations driven by the menstrual cycle, especially regarding sex hormones like 17 β-estradiol, which might influence inflammatory and oxidative stress responses. To capture a comprehensive and reliable profile of each participant’s baseline status, repeated sampling across different cycle phases was essential to improve knowledge about female physiology. The single HIIT session, placed strategically two hours after baseline assessments, served as a controlled physiological stressor to probe the acute response mechanisms.

### 2.2. Pretesting

The pretesting phase consisted of two separate screening appointments. On the first day, body composition was assessed using bioelectrical impedance analysis (BIA), a resting electrocardiogram (EKG) was conducted and participants underwent a familiarization session to ensure correct execution of the resistance exercises. On the second day, venous blood samples were collected to assess general health and screen for any indicators of underlying disease. Additionally, a 5-repetition maximum (5-RM) test was performed, allowing for estimation of each participant’s individual one-repetition maximum (1-RM) using the Brzycki formula [24]. This was essential for determining the appropriate intensity, which was defined as 50% of 1-RM for the HIIT session on the intervention day. A washout period of at least seven days was implemented between each screening session and experimental trial to ensure full recovery of blood biomarkers and to minimize the influence of residual muscle soreness on the study outcomes.

### 2.3. Nutrition and Physical Activity

Study participants were instructed to maintain their habitual dietary patterns and physical activity throughout the intervention period, including the main trial day and the seven baseline blood sampling sessions. They were required to refrain from taking any nutritional supplements and to limit physical activity to a maximum of two hours per week in order to minimize exercise-induced adaptations that could potentially have affected study outcomes.

Prior to each appointment, participants were asked to abstain from alcohol and any physical activity for at least 48 h and to undergo an overnight fast of 12 h. On the morning of testing, participants were instructed to consume 0.5 L of water to ensure standardized hydration status. To maintain consistency across testing conditions, they were also advised to travel to the study site either on foot or via public transport.

### 2.4. Experimental Protocol

This study was registered on clinicaltrials.org (NCT05242978) and it was approved by the Ethics Committee of the University of Vienna, Austria (ID 00743—1.12.2021). Study participants received information about the aim, risks and experimental procedures and gave their written consent before enrollment into the study.

Each participant arrived at the study center at 8:30 a.m. On seven occasions, a single set of blood samples was collected, consisting of one venous sample (serum and plasma) and one capillary sample. On one of these occasions, participants performed a HIIT session two hours after the initial blood collection. On this intervention day, further blood samples were collected immediately before the exercise session (pre-HIIT), immediately after (post-HIIT) and after 15 min of recovery (15-post-HIIT).

Capillary blood was obtained from the fingertip and immediately used for detection of reactive oxygen species (ROS) via electron paramagnetic resonance (EPR) spectroscopy. Venous blood samples were drawn into EDTA tubes, centrifuged at 3500 rpm for 15 min and the resulting plasma was aliquoted and stored at −80 °C for subsequent biochemical analysis.

The six baseline assessments were conducted with the aim of obtaining as many and as diverse estradiol levels as possible from each participant across different phases of the menstrual cycle. To ensure broad representation across the menstrual cycle despite the time-intensive nature of exercise testing, participants were deliberately selected to capture a wide range of cycle phases.

On each testing day, estradiol concentrations were measured and the menstrual cycle day was determined using the calendar method, based on the participant’s self-reported first day of menstruation and self-reported average cycle length. The calendar method to determine menstrual cycle phases has been commonly used in research [25,26,27,28,29]. Menstrual cycle phase was determined using a validated low-burden questionnaire [30] to classify participants as being in either the follicular or luteal phase. In addition, participants were required to prospectively self-track their menstruation on a daily basis throughout the entire study period. This combined approach was implemented to enhance the accuracy and reliability of menstrual cycle phase classification. We wanted the study to be cost-efficient and feasible. Furthermore, a menstruation “yes” classification was used if a participant reported active menstrual bleeding on the testing day. Nevertheless, it is recommended for future trials to additionally measure basal body temperature and serial estradiol measurements to further strengthen the accuracy of menstrual cycle classification.

This approach aimed to collect a comprehensive dataset of estradiol levels and menstrual cycle information from the same participants across different time points.

This dataset is intended to be a role model for future clinical trials in sports science or related disciplines by showing an opportunity for performing confounding analyses involving hormonal fluctuations and menstrual cycle phases, addressing a common limitation in research where female participants are often underrepresented due to the complexity of controlling for menstrual cycle-related variables and hormones such as estradiol.

On one of their study visits, each participant additionally had to perform one HIIT session, the procedure of this intervention day will now be explained in more detail.

### 2.5. HIIT Protocol

Study participants completed a single session of HIIT. Prior to each session, participants were instructed to rest for two hours and to consume 0.5 L of tap water to ensure a consistent hydration status. Training sessions commenced at 11:00 a.m. and were supervised by a sports scientist. To standardize exercise intensity, all exercises were performed at 50% of the participant’s individual one-repetition maximum (1-RM), with a controlled speed of one second for both concentric and eccentric phase. Individual 1-RM values were calculated using the Brzycki [24] formula, based on a 5-RM test conducted at least seven days prior to the intervention.

The resistance circuit included rowing, chest press, leg curl, lat pulldown and leg press. Each exercise was performed for 40 s, followed by a 10 s rest period before initiating the next exercise. Participants completed three rounds of the circuit, with 60 s rest intervals between circuit one and two and between two and three. The exercise protocol was specifically designed to elicit an acute physiological stress response.

The structure of the HIIT protocol was inspired by the protocols by Bizheh and Zheng, which demonstrated an acute increase in inflammatory markers and ROS [19,20]. Subjective perception of exercise intensity was monitored using the BORG scale, ranging from 6 (resting state) to 20 (maximal intensity), with a target minimum score of 15 to ensure sufficient exercise intensity. Participants were instructed to report their perceived level of exertion immediately before exercise as well as after exercise.

### 2.6. Laboratory Methods

**Reactive oxygen species (ROS)** formation was detected using EPR (Bruker, Ettlingen, Germany). At each time point, 12.5 µL of capillary blood was mixed with an equal volume of oxygen-sensitive label (Noxygen, Elzach, Germany, NOX15.1-5 µmol/L) and 25 µL of Krebs-HEPES buffer-diluted CMH spin probe (400 µmol/L). A 40 µL aliquot was transferred into a capillary tube (Hirschmann, Eberstadt, Germany) and incubated for 60 s at 37 degrees Celsius, using a temperature and gas controller (Noxygen, Elzach, Germany). The EPR settings included: center field: 3489.5 G, sweep width: 60.0 G, and frequency: 9.779333 GHz, with ROS levels expressed in µmol/min.

**Plasma Interleukin-6 (IL-6)** concentrations were quantified using the human IL-6 high-sensitivity ELISA kit (Thermo Fisher Scientific, Waltham, MA, USA, BMS213—mean detectable concentration 6.4 pg/mL).

**Estradiol** concentrations (pg/mL) were measured in serum using a chemiluminescent microparticle immunoassay.

### 2.7. Statistical Analysis

Sample size estimation was performed using G-Power 3.1 (Windows 10), with an effect size of 0.33 and a power level of 0.9, which resulted in a required sample of 30 subjects. All statistical analyses as well as all figures were performed and generated using SPSS (version 28.0, IBM, New York, NY, USA). The results of the descriptive analyses are presented as mean +/− standard deviation (SD).

A normal distribution was demonstrated with the Kolmogorov–Smirnov test and dependent *t*-tests elaborated changes over time. As a requirement for the Kolmogorov–Smirnov test, Levene’s test was performed to test the variance of homogeneity. Continuous data without a normal distribution were log-transformed via Johnson transformation. Evaluation of differences between groups (luteal vs. follicular phase, menstruation yes vs. no, estradiol low vs. high) were conducted using independent *t*-tests. To assess within-subject changes over time (from baseline to 15-post-HIIT), a repeated-measures ANOVA was employed. Where applicable, Mauchly’s test was used to assess the assumption of sphericity and Greenhouse–Geisser corrections were applied if this assumption was violated. The statistical significance was set at *p* < 0.05. The data are reported as mean ± standard deviation unless otherwise specified. All tests were two-sided. Outliers were eliminated if they were above the 3rd standard deviation.

The cut-off value of 72.5 pg/mL (mean estradiol level of the study population) was used to dichotomize estradiol concentrations into “low” (*n* = 104; mean estradiol concentration: 38.77 ± 20.00 pg/mL) and “high”(*n* = 104; mean estradiol concentration: 151.29 ± 62.99 pg/mL) estradiol. Estradiol concentrations were significantly higher in group “high-estradiol” compared to group “low-estradiol” (38.77 ± 20.00 vs. 151.29 ± 62.99 pg/mL using a two-sided *t*-test (*p* < 0.001).

## 3. Results

### 3.1. Results of Cross-Sectional Study

Results from the cross-sectional part of the study are presented in Table 1.

No significant differences were found between estradiol levels (low (11–≤72.5 pg/mL) vs. high (>72.5–394 pg/mL)): −0.038 µmol/min (*p* = 0.266), menstrual cycle phase (luteal vs. follicular; 0.013 µmol/min (*p* = 0.716)) and menstruation status (yes vs. no: −0.056 µmol/min (*p* = 0.259)) for baseline ROS were observed.

No significant differences between 17 β-estradiol levels (low vs. high: +0.015 pg/mL (*p* = 0.906)) for baseline IL-6 concentrations were observed.

Further, no significant differences between menstrual cycle phase (luteal vs. follicular −0.052 (*p* = 0.679)) for baseline IL-6 concentrations were observed.

Subjects without current menstruation showed a tendency of lower baseline IL-6 concentrations than subjects with acute menstruation (yes vs. no: −0.302 pg/mL; *p* = 0.088).

### 3.2. Results Intervention Day (n = 30)

The changes in ROS and IL-6 levels from baseline to 15-post-HIIT are presented in Table 2 and Table 3.

No significant differences between estradiol concentrations (ROS: *p* = 0.477; IL-6: *p* = 0.249), menstrual cycle phase (ROS; *p* = 0.752; IL-6: *p* = 0.557) or menstruation status (ROS: *p* = 0.383; IL-6: *p* = 0.808) with baseline, pre-HIIT, post-HIIT or 15-post-HIIT ROS and IL-6 levels were observed.

The relationship between menstrual cycle phase, menstruation status, estradiol level and exercise performance is presented in Table 4.

No significant differences between menstrual cycle phase (*p* = 0.990), menstruation status (*p* = 0.605) and 17 β-estradiol level (*p* = 0.653) and exercise performance using independent *t*-tests were observed.

No significant differences between menstrual cycle phase (*p* = 0.720), menstruation status (*p* = 0.522) and 17 β-Estradiol level (*p* = 0.726) and exercise-induced % change in BORG, using independent *t*-tests were observed, as shown in Table 5.

## 4. Discussion

### 4.1. Summary of the Main Findings

The present study investigated the association between endogenous 17-beta-Estradiol concentrations, menstrual cycle phase and menstruation status with exercise-induced ROS formation, IL-6 responses and strength performance following a single bout of resistance-based high-intensity interval training (HIIT) in young healthy female adults. Contrary to our hypothesis, neither menstrual cycle phase nor circulating estradiol levels significantly influenced baseline or exercise-induced ROS and IL-6 responses, nor HIIT performance outcomes.

### 4.2. Mechanistic Interpretation

Exercise-induced inflammation and ROS formation remain complex and sometimes controversial topics in sports science. IL-6 exhibits a dual role, acting as a pro-inflammatory cytokine during acute exercise while also exerting anti-inflammatory effects [31]. Similarly, ROS function as signaling molecules that promote adaptive processes such as mitochondrial biogenesis and antioxidant defense, although excessive ROS may result in oxidative damage and impaired cellular function [32]. Moderate increases in IL-6 and ROS are therefore considered beneficial for physiological adaptation, whereas chronic or excessive elevations may contribute to inflammation and oxidative stress-related pathologies [33].

Although estradiol has been proposed to possess antioxidative properties, these effects did not translate into measurable differences in ROS or IL-6 in the present study. Mechanistically, estradiol may exert protective effects through both direct and indirect pathways, including free-radical scavenging due to its phenolic structure, thereby reducing lipid peroxidation and ROS-induced cellular damage [5,34]. However, the physiological fluctuations of estradiol across the menstrual cycle may be too modest to induce detectable systemic changes in oxidative stress or inflammatory markers under the conditions of acute resistance-based HIIT.

### 4.3. Contextualization with the Existing Literature

Our findings are consistent with previous studies from Meignié and Colenso-Semple, who reported no substantial effects of menstrual cycle phase on strength performance or post-exercise recovery markers [11,18].

In contrast, a systematic review and meta-analysis suggested that exercise performance may be slightly impaired during the early follicular phase compared to other phases [35]. Additionally, studies reporting reduced strength or increased delayed onset muscle soreness (DOMS) during the early follicular phase [16] may reflect differences in exercise modalities, sample characteristics or methodological approaches.

Furthermore, it is important to note that during the luteal phase, high-intensity intermittent exercise may modulate cytokine responses and facilitate recovery in healthy women [13].

The absence of significant differences in our study may indicate that local muscular and immune–metabolic responses to resistance exercise are robust enough to override normal physiological hormonal fluctuations, particularly in the absence of extreme exercise stimuli or pathological hormonal conditions.

### 4.4. Potential Explanations for the Null Findings

Several factors may explain the lack of observational effects. First, the magnitude of estradiol fluctuations may not have been sufficient to induce measurable systemic changes in ROS or IL-6. Second, systemic biomarkers may not adequately reflect localized muscular oxidative stress or inflammatory responses.

Additionally, the exercise stimulus, a single session of resistance-based HIIT, may not have been strong or prolonged enough to reveal subtle hormonal modulation. Differences in study design compared to previous work, such as the protocol by Pal [6], may also contribute. In that study, female participants were tested during a narrow window (10 to 13 days after menstruation onset) to capture peak estradiol levels, whereas the present study used a broader and less restrictive design. While this increases ecological validity, it may reduce sensitivity to detect hormone-related effects.

### 4.5. Study Limitations

Several limitations should be acknowledged. The study population consisted of healthy, sedentary females with low-to-moderate physical activity levels, limiting generalizability. Training status is known to influence ROS and IL-6 responses, with trained individuals typically showing lower ROS production and a blunted IL-6 response due to enhanced antioxidant capacity and improved inflammatory regulation [36,37], whereas untrained individuals tend to exhibit greater responses [33].

The study design included only a single exercise session, limiting intra-individual comparisons. Menstrual cycle phase classification relied on self-reported data and the calendar method, which is not the gold standard. The dichotomization of estradiol levels using a cut-off value of 72.5 pg/mL may not accurately reflect physiologically meaningful thresholds.

Furthermore, the small sample size of the “menstruation yes” subgroup further limits statistical power and reduces the ability to detect subtle subgroup-specific effects. Finally, although participants were instructed to maintain consistent lifestyle habits, the lack of objective monitoring of sleep, together with the fasted exercise condition, may have introduced additional variability in metabolic and inflammatory responses.

### 4.6. Implications and Future Directions

These findings suggest that in healthy young women, natural fluctuations in estradiol and menstrual cycle phase may not meaningfully influence systemic oxidative stress, inflammatory responses or performance following resistance-based HIIT.

Future research should incorporate more detailed assessments of local muscle responses, such as muscle biopsies or tissue-specific measurements to better capture potential localized effects of hormonal fluctuations.

Moreover, including trained populations and implementing repeated intervention sessions may further clarify the interaction between hormonal status, exercise adaptation and performance outcomes.

## 5. Conclusions

In conclusion, this study supports the notion that neither menstrual cycle phase nor estradiol levels are significant confounders for exercise-induced ROS and IL-6 formation and exercise performance in young healthy women.

These findings advocate for greater inclusion of female subjects in sports science without the need to exclude participants based on hormonal status in non-clinical settings. Nevertheless, we recommend that future studies involving female participants consistently measure sex hormones such as 17-β estradiol and the further improvement of menstrual cycle phase determination via measuring basal body temperature [38] to further increase knowledge in this under-investigated field of life sciences. Incorporating the assessment of variables such as menstrual cycle phase and estradiol levels in clinical trials investigating oxidative stress and inflammation-related topics in female participants would facilitate high-quality research and improve the reliability of sex-specific analyses.

## Figures and Tables

**Table 1:** Association of estradiol levels and menstrual cycle characteristics with baseline ROS and IL-6 levels.

Variable	Baseline-ROS (µmol/min)	Baseline IL-6 (pg/mL)
Estradiol 11–≤72.5 pg/mL (*n* = 104)	0.99 ± 0.23	1.36 ± 0.89
Estradiol > 72.5–384 pg/mL (*n* = 104)	1.03 ± 0.26	1.38 ± 0.90
Follicular phase (*n* = 106)	1.02 ± 0.26	1.34 ± 0.91
Luteal phase (*n* = 101)	1.01 ± 0.25	1.39 ± 0.89
Menstruation no (*n* = 180)	1.01 ± 0.25	1.32 ± 0.89
Menstruation yes (*n* = 30)	1.01 ± 0.26	1.63 ± 0.94

**Table 2:** Changes in ROS from baseline to 15-post-HIIT. Comparisons between estradiol concentrations, menstrual cycle phase and menstruation status. Statistical differences were measured using RMANOVA.

Variable	Baseline ROS	Pre-HIIT ROS	Post-HIIT ROS	15-Post-HIIT ROS	RMANOVA (4 × 6)	
					Time	Group
**Estradiol**					*p* < 0.05	*p* = 0.477
E low (14)	0.91 ± 0.14	0.89 ± 0.19	1.02 ± 0.28	0.90 ± 0.19		
E high (15)	1.05 ± 0.30	0.95 ± 0.22	1.06 ± 0.31	0.99 ± 0.25		
**Cycle Phase**					*p* = 0.21	*p* = 0.752
FP (14)	0.95 ± 0.20	0.91 ± 0.21	1.04 ± 0.28	0.92 ± 0.20		
LP (14)	1.04 ± 0.28	0.94 ± 0.21	1.05 ± 0.32	0.98 ± 0.25		
**Menstruation**					*p* < 0.05	*p* = 0.383
M no (26)	0.98 ± 0.25	0.92 ± 0.22	1.02 ± 0.29	0.93 ± 0.21		
M yes (4)	0.92 ± 0.21	0.84 ± 0.18	1.05 ± 0.33	0.99 ± 0.28		

RMANOVA (4 × 6): four (time) × 6 (condition) repeated-measures ANOVA. Time: Time effect across the four measurement points. Group: Group effect comparing estradiol categories/menstrual phase/menstruation status. HIIT: High-intensity interval training. ROS: Reactive oxygen species. E low: Estradiol 11.0–≤71.0 pg/mL. E high: Estradiol >71.0–283.0 pg/mL. FP: Follicular phase. LP: Luteal phase. M no: No current menstruation. M yes: Current menstruation.

**Table 3:** Changes in Interleukin-6 (IL-6) from baseline to 15-post-HIIT. Comparisons between estradiol concentrations, menstrual cycle phase and menstruation status. Statistical differences were measured using RMANOVA.

Variable	Baseline IL-6 (pg/mL)	Pre-HIIT IL-6	Post-HIIT IL-6	15-Post-HIIT IL-6	RMANOVA (4 × 6)	
					Time	Group
**Estradiol**					*p* < 0.001	*p* = 0.249
E low (14)	1.67 ± 1.06	1.23 ± 0.89	1.99 ± 1.22	1.45 ± 0.74		
E high (14)	1.14 ± 0.79	1.17 ± 0.62	1.84 ± 1.26	1.51 ± 0.78		
**Cycle Phase**					*p* < 0.05	*p* = 0.557
FP (14)	1.55 ± 1.05	1.20 ± 0.85	1.80 ± 1.11	1.37 ± 0.70		
LP (14)	1.16 ± 0.81	1.15 ± 0.67	1.89 ± 1.29	1.54 ± 0.80		
**Menstruation**					*p* < 0.05	*p* = 0.808
M no (26)	1.29 ± 0.91	1.12 ± 0.62	1.80 ± 1.21	1.42 ± 0.73		
M yes (4)	1.80 ± 1.25	1.45 ± 1.48	2.26 ± 1.48	1.58 ± 1.09		

RMANOVA (4 × 6): four (time) × 6 (condition) repeated measures. Time: Time effect across the four measurement points. Group: Group effect comparing estradiol categories/menstrual phase/menstruation status ANOVA. HIIT: High-intensity interval training. IL-6: Interleukin-6. E low: Estradiol 11.0–≤71.0 pg/mL. E high: Estradiol >71.0–283.0 pg/mL. FP: Follicular phase. LP: Luteal phase. M no: No current menstruation. M yes: Current menstruation.

**Table 4:** Exercise performance outcomes across menstrual cycle phase, menstruation status and estradiol level.

Variable	Exercise Performance (kg)	*p* Value
**Menstrual Cycle Phase**		*p* = 0.990
Follicular phase (14×)	10.957 ± 2.111	
Luteal phase (14×)	11.034 ± 2.432	
**Menstruation Status**		*p* = 0.605
No (26×)	10.901 ± 2.273	
Yes (4×)	11.517 ± 1.380	
**Estradiol**		*p* = 0.653
11.0–≤71.0 pg/mL (14×)	10.766 ± 1.957	
>71.0–283.0 pg/mL (15×)	11.144 ± 2.464	

**Table 5:** Menstrual cycle phase, menstruation status and estradiol level and BORG scale.

Variable	BORG % Change Pre-HIIT to Post-HIIT	*p* Value
**Menstrual Cycle phase**		*p* = 0.720
Follicular phase (14×)	185.7 ± 39.1	
Luteal phase (14×)	175.2 ± 34.1	
**Menstruation Status**		*p* = 0.522
No (26×)	179.3 ± 36.9	
Yes (4×)	191.7 ± 28.9	
**Estradiol**		*p* = 0.726
11.0–≤71.0 pg/mL (14×)	183.3 ± 40.8	
>71.0–283.0 pg/mL (15×)	178.5 ± 31.8	

## Data Availability

All data is contained within the article.
